# Leiomyoma: An Exceptional Benign Tumor of the Kidney

**DOI:** 10.7759/cureus.66519

**Published:** 2024-08-09

**Authors:** Karich Nassira, Younesse Najioui, Anass Haloui, Tijani El Harroudi, Amal Bennani

**Affiliations:** 1 Pathology, Mohammed VI University Hospital, Faculty of Medicine and Pharmacy of Oujda, Mohammed First University of Oujda, Oujda, MAR; 2 Pathology, Mohammed VI University Hospital, Oujda, MAR; 3 Surgical Oncology, Mohammed VI University Hospital, Regional Oncology Center, Oujda, MAR; 4 Pathology, Mohammed VI University Hospital, Faculty of Medicine and Pharmacy of Oujda, Mohammed First University, Oujda, MAR

**Keywords:** case report, benign tumor, surgery, leiomyoma, kidney neoplasm

## Abstract

Renal leiomyoma is a benign finding in kidney pathology. It has been documented in various organs; renal location is less frequent and has been rarely documented in the literature. We present here the case of a renal leiomyoma revealed by an abdominal mass and flank pain. The diagnosis of certainty is histological, generally on surgical specimens. Due to the challenges associated with clinically diagnosing this tumor, a high level of suspicion is warranted when a patient presents with sizable and clearly defined renal lesions.

## Introduction

Renal leiomyomas are uncommon, non-cancerous tumors in the kidney that originate from smooth muscle cells [1]. The initial documentation of a renal leiomyoma dates back to 1890 [2]. A majority of leiomyomas, around two-thirds, are observed in women [3]. The distinction between leiomyomas and other malignant tumors is achievable through histological examination. Unlike aggressive tumors, renal leiomyomas typically exhibit non-metastatic behavior. Following surgical intervention, the prognosis is highly favorable, with a low risk of recurrence [1]. We present a case involving a 55-year-old woman who experienced a palpable mass of the right kidney, measuring 16 cm in length, and flank pain, ultimately diagnosed with leiomyoma.

## Case presentation

**Figure 1:**
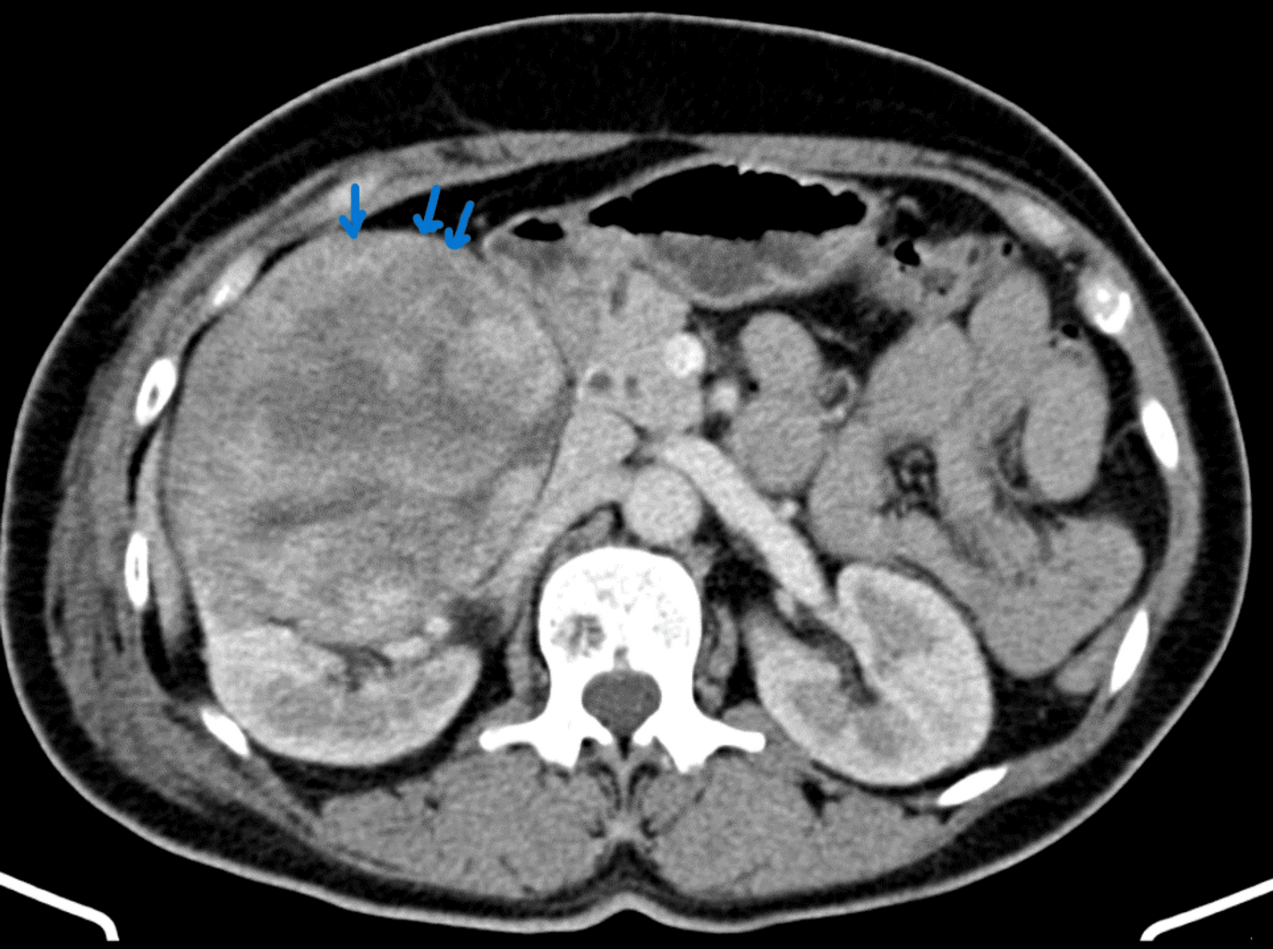
CT slice showing a well circumscribed mass of the right kidney of heterogeneous density (blue arrows).

A 55-year-old menopausal female patient presented to the digestive surgery department for palpable mass and right flank pain. She has a history of breast carcinoma, for which she has undergone breast resection, lymph node dissection, and chemotherapy. Urinalysis revealed microscopic haematuria. Serum creatinine was normal. A physical examination on admission revealed palpable masses that were painful, without weight loss or other accompanying symptoms. Computerized tomography (CT) of the abdomen showed a well-circumscribed mass (16.5 cm × 12 cm × 8.5 cm) of heterogeneous density abutting the entire right kidney (Figure 1).

**Figure 2:**
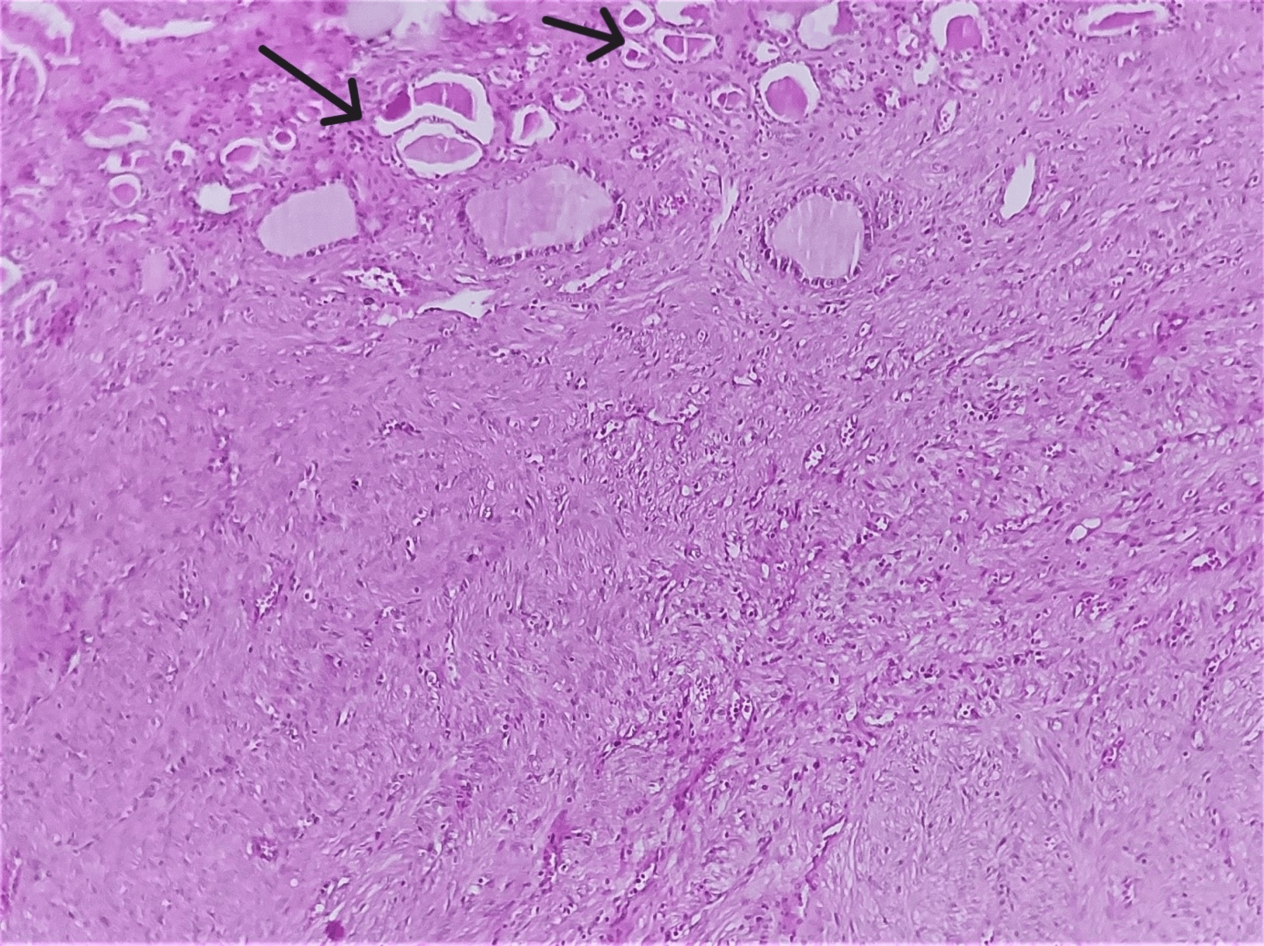
Histological image showing the renal parenchyma (black arrows) with fusocellular proliferation devoid of atypia and mitosis (HE x100).

The patient underwent a right radical nephrectomy. On gross examination of the specimen, we note the presence of a lesion appended to the renal apex, measuring 12 cm × 7.5 cm × 6 cm of white-grey colour, well limited without hemorrhagic remodelling. Microscopically, it was composed of interlacing fascicles of spindle cells without mitotic figures or pleomorphism (Figure 2).

**Figure 3:**
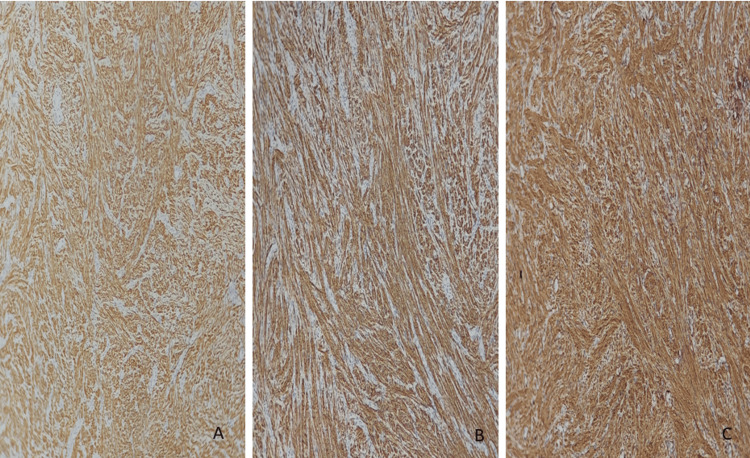
Micrographic image showing positive labeling of tumor cells by smooth muscle markers. (A) SMA, (B) desmin, and (C) H-caldesmon (IHC × 100).

The immunohistochemical study of the cells proved positive for actin, H-caldesmon, and desmin and negative for cytokeratin, S-100, and HMB-45 (Figure 3). Overall, the diagnosis of renal leiomyoma was retained. After one year of follow-up, the patient was found to be asymptomatic and free of disease.

## Discussion

Postmortem observations of renal leiomyoma, a rare and benign tumour, approximate a rate of 5%. Renal leiomyomas constituted 1.5% of benign renal tumours and only 0.3% of all treated renal umors. Incidentally found in most cases, these tumours are most commonly reported in adult women in the fourth decade [3-5].

The origin of renal parenchymal leiomyoma remains unclear due to the tumour's rarity. It is hypothesized to originate from the smooth muscles of blood vessels or renal stem cells. Clinically, renal leiomyomas are often asymptomatic. When symptoms occur, they can manifest as an abdominal palpable mass, abdominal pain, or rarely as hematuria. Imaging studies alone are insufficient for an accurate diagnosis of kidney leiomyoma [6,7].

Based on clinical features, two groups of leiomyomas are described: small, asymptomatic tumours, sometimes multifocal and often incidentally discovered, and large types, usually solitary. However, asymptomatic, large forms of leiomyomas exist [8]. Macroscopically, renal leiomyomas are well-defined, peripherally located and have a solid and elastic consistency with a white or red tan. Necrosis and invasion of adjacent structures are common findings in leiomyosarcomas, contrary to leiomyomas [9].

Histologically, renal leiomyomas consist of spindle cell proliferation without atypia or mitotic figures; hyperchromasia, pleomorphism, or soft tissue invasion are indicative of leiomyosarcoma [2,10]. The main differential diagnosis includes spindle cell tumours in the retroperitoneum, such as angiomyolipoma, neurofibromas, and schwannomas. Immunohistochemical markers, specifically smooth muscle antigens like specific smooth-muscle actin and desmin, aid in distinguishing leiomyomas from other tumours [11].

Surgery, particularly radical nephrectomy, remains the gold standard treatment for this entity, with no need to add further adjuvant therapy as the prognosis is favourable [4].

## Conclusions

Renal leiomyomas are benign tumours with non-aggressive behaviour and no tendency to metastasize. A heightened level of suspicion is warranted when a patient exhibits large, clearly defined renal lesions, particularly those located in the peripheral capsular region. Following surgery, specifically a radical nephrectomy, the prognosis is highly favourable with no recurrence. This underscores the challenge of clinically differentiating a leiomyoma from other malignant lesions, emphasizing the necessity of histological examination for an accurate differential diagnosis.
